# Efficiency of Osmotic Dehydration of Apples in Polyols Solutions

**DOI:** 10.3390/molecules23020446

**Published:** 2018-02-17

**Authors:** Joanna Cichowska, Joanna Żubernik, Jakub Czyżewski, Hanna Kowalska, Dorota Witrowa-Rajchert

**Affiliations:** Department of Food Engineering and Process Management, Warsaw University of Life Sciences, Nowoursynowska 166, 02-787 Warszawa, Poland; joanna_zubernik@sggw.pl (J.Z.); jakub_czyzewski@sggw.pl (J.C.); hanna_kowalska@sggw.pl (H.K.); dorota_witrowa_rajchert@sggw.pl (D.W.-R.)

**Keywords:** osmotic dehydration, polyols, apple, water loss, solid gain

## Abstract

The present study aimed to evaluate the influence of selected compounds from the polyol group, as well as other saccharides, on the osmotic dehydration process of apples. The following alternative solutions were examined: erythritol, xylitol, maltitol, inulin and oligofructose. Efficiency of the osmotic dehydration process was evaluated based on the kinetics of the process, and through comparison of the results obtained during the application of a sucrose solution. This innovative research utilizes alternative solutions in osmotic pretreatment, which until now, have not been commonly used in fruit processing by researchers worldwide. Results indicate that erythritol and xylitol show stronger or similar efficiency to sucrose; however, the use of inulin, as well as oligofructose, was not satisfactory due to the insufficient, small osmotic driving forces of the process, and the low values of mass transfer parameters.

## 1. Introduction

Osmotic dehydration (OD), as pre-treatment before drying, is commonly used to preserve food, and allows modification of the composition and properties of the products, as well as an opportunity to create new food products and innovative technologies. Nowadays, OD receives a lot of attention in the field of fruit preservation, as it allows for improvements in food product quality and reduces energy consumption. Efficiency of OD depends on many factors, such as duration of the process, temperature, solution concentration and type [1], the material structure (porosity), the sample geometry (size, shape, and surface area), and the ratio of the mass of the food to the mass of the solution [2]. A number of osmotic agents can be used in OD. The osmotic agent must be effective, convenient, non-toxic, taste good, and should be readily dissolvable to form a high-concentrated solution [3]. In order to select the appropriate substance, it is necessary to take its molecular weight into account. During osmosis, the kinetic parameters are strongly affected by the kind of osmotic agent used, including its molecular weight and ionic behavior [4]. The osmotic pressure of solutions depends on the concentration of low-molecular-weight substances [5]. High-molecular-weight substances produce a lower osmotic pressure, resulting in lower kinetic parameters, and less penetration of the high-molecular-substances into the material [6]. Various substances can be used for the preparation of such solutions: glucose, sucrose, glycerol, sorbitol, corn syrup, glucose syrup, and fructooligosaccharide are most commonly used [7]. Most of the papers dealing with osmotic treatment of foodstuffs have mainly focused on dehydration in quite popular solutions. Researcher have reported the influence of OD on process kinetics in, for example, sucrose [8,9,10], glucose, fructose [11] and trehalose [12,13]. Fewer studies are concerned with the effects of using an alternative solution to sucrose substances, such as sorbitol [14,15,16], maltose [17], oligofructose [16,18] and maltodextrin [15,18]. Researchers have also utilized non-conventional osmotic agents, including maltitol [13,19], glycerol [15,20], polydextrose [15], galaktosorbitol [16], and tagatose [21].

In this study, selected compounds from the polyol group, as well as other alternative saccharides, were used for pre-treatment. The present study aimed to evaluate the influence of erythritol, xylitol, maltitol and oligofructose in pre-treatment, on the OD of apples, as alternatives to sucrose. Observation of the kinetic parameters during the process is essential for the selection of the optimal processing condition.

## 2. Results and Discussion

### 2.1. Water Content (WC)

The values of WC decreased with the prolongation of the dehydration process. Table 1 and Table 2 shows the changes in the WC during the experiments. The raw material was characterized by a WC of 7.31 ± 0.01 g/g d.m. In all cases, an increase in the osmotic solution concentration resulted in a greater degree of dehydration of the apples. The highest degree of dehydration was achieved using a 40% solution concentration of xylitol and erythritol–WC was reduced to about 1.60 g/g d.m.; therefore, these alternative solutions are more effective compared to the 50% solution concentration of sucrose. A 40% solution concentration of maltitol, and a 30% solution concentration of erythritol showed similar efficiency to sucrose. The use of solutions containing inulin and oligofructose, as well as a 20% solution of maltitol, were found to be ineffective. Even though statistical analysis indicated an influence of the type of osmotic agent (Table 3), one-way ANOVA did not show any significant influences of time or concentration on WC values, when inulin and oligofructose solutions were used. The larger osmotic effect noted with xylitol and erythritol solutions, compared with sucrose, has been attributed to the higher (almost double) molecular weight of sucrose and the resultant lower osmotic pressure, compared to the alternative solutions. 

In recent research, Rodriguez et al. [22] dehydrated nectarines for 2 h (with an initial WC of 4.602 g/g d.m.), in a 60% solution concentration of sorbitol to achieve a reduction of WC to a value of 1.903 ± 0.779 g/g d.m., whereas a 40% solution concentration of glucose resulted in poor reduction of WC values. Moreover, when glucose and sorbitol were used (2.52 g/g d.m. and 2.59 g/g d.m., respectively), the resulting values were similar to findings in the present study, which utilized the same conditions: a 2-h duration with a 40% solution concentration of xylitol and maltitol (Table 1). Brochier et al. [15] dehydrated yacon in a 33% concentration of alternative solutions to sucrose, namely, maltodextrin, polydextrose, sorbitol and glycerol, for 30, 60, 120, 240 and 360 min. Their results confirmed that a decrease of WC was observed during the process. The best results were achieved when glycerol and sorbitol were used. Maltodextrin did not behave like an osmotic agent in their study.

### 2.2. Water Loss (WL)

The WL is an important parameter of mass transfer that indicates the efficiency of OD. To evaluate the acceptability of utilizing alternative solutions, the curves of OD kinetics in the 50% sucrose solution were applied to the figures (shown below as dotted lines).

**Figure 1:**
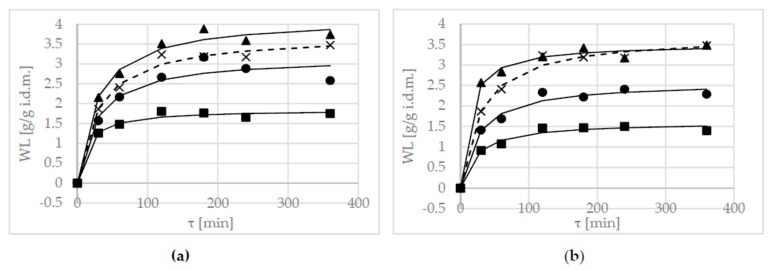

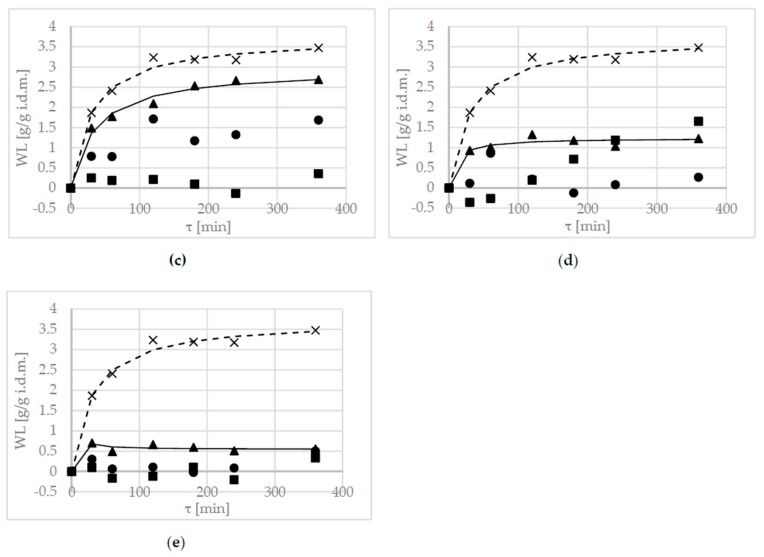
Water loss (WL) kinetics at several concentrations, (20 (■), 30 (●), 40 (▲) and 50 (×) g/100g) at 40 °C, in different solutions: (**a**) erythritol, (**b**) xylitol, (**c**) maltitol, (**d**) inulin, (**e**) oligofructose. Lines are the Peleg’s model. The dotted line is the kinetic reference (sucrose).

The non-linear increase of WL was observed at all concentrations during the OD process. In all cases, except for solutions with oligofructose, inulin, and maltitol, at 20% concentrations an initial rapid rate of mass transfer was observed, followed by a decrease in this rate (Figure 1). This indicates that the system was approaching the end of the osmotic process (i.e., pseudo equilibrium) [10].

This phenomenon was most pronounced for the first three hours of the process. In this study, osmotic pre-treatments for periods longer than 3 h were not effective. This relationship was the best demonstrated in the case of erythritol (Figure 1a) and xylitol (Figure 1b) solutions. This suggests that it is not necessary to dehydrate the apple slices in solutions for periods longer than 3 h. Statistical analysis also confirmed this statement—values achieved after 180 min or longer were classified into one homogeneous group (Table 5).

Brochier et al. [15] observed that the change in moisture content was no longer significant after one hour with the use of glycerol, sorbitol or polydextrose. A high rate of WL at the beginning of the process was reported in research by Moreira et al. [20] during OD of chestnut, as well as in research by Khan et al. [23] during OD of apples. This is due to the difference in osmotic pressure between the osmotic solution and the food. Subsequently, the phenomenon decreases because the concentration gradient between the solution and the food decreases over-time [2]. 

Rizzolo et al. [24] dehydrated strawberry slices in 60% concentrated solutions of sucrose and sorbitol for 6 h at a temperature of 30 °C. They observed that WL had constantly increased during both of the processes. Higher values were obtained when the sorbitol solution was used. This fact was due to the different molecular weights of sorbitol (182.18 g/mol) and sucrose (342.30 g/mol), which, at the same concentrations, yielded different water activities (0.87 and 0.93, respectively). 

In this research, OD in a 40% solution concentration of erythritol and xylitol was more effective compared to the dehydration process in a sucrose solution (Figure 1a,b). This could also be explained by the lower molecular weight of erythritol and xylitol (122.12 g/mol and 152.15 g/mol, respectively). 

The increase in solution concentration resulted in an increase of the osmotic pressure gradients and, hence, higher WL in all cases (except in solutions which contained inulin and oligofructose) (Figure 1d,e). Similar results were reported by Khan et al. [23] in the OD of apples in 40% to 60% solution concentrations, and by Djendoubi et al. [25] who carried out the OD process of pears in a sucrose solution (from 25 to 65^o^Brix). This is explained by an increase in the osmotic driving force between the sample and the surrounding solution. The increase in temperature decreases the viscosity of the osmotic solution, and the resistance to the mass transfer between the surface and the osmotic solution, thus facilitating the outflow of water from the sample, and the diffusion rate of solute into the sample [2]. The influence of temperature on OD was confirmed by Devic et al. [26], in the OD of apples at temperatures of 45 °C and 60 °C.

In this research, all of the concentrations (20% to 40%) of maltitol solutions were less effective compared to sucrose as the solute (Figure 1c). The higher concentration of maltitol (50%) resulted in higher values of WL and solid gain (SG), compared to sucrose [27]. In a few research studies, the Peleg’s equation has been often used to model the kinetics of WL and solute uptake during OD [10,28,29]. 

To evaluate the goodness of fit of the models in different solutions, parameter values of modeling WL using Peleg’s model are shown in Table 4. The use of Peleg’s modeling of OD was effective in all of the concentrations of erythritol and xylitol solutions—the goodness of fit has high R^2^ values, and low RMSE and χ^2^ values (Table 4). In the case of other solutions, modeling WL using Peleg’s model was effective only at 40% concentrations—in the other cases, values for parameter CRV were more than 20%, which indicates that the model could not be used for the prediction of WL.

The k_1_ relates to the dehydration rate at the very beginning of the process. The reciprocal of k_1_ describes the initial mass transfer rate (i.e., the lower the k_1_, the higher the mass transfer rate) [10]. It can be seen from the data in Table 4, that at constant temperature, k_1_ decreased with increased solution concentrations from 20% to 40%, which indicates an increase in the initial rate of mass transfer terms (the highest value was observed in the 40% solution concentration of xylitol).

It has been shown [30] that the k_2_ parameter defines the equilibrium moisture content (and soluble solids)—a value that is expected to vary with the syrup concentration. These results are not surprising. The lower the k_2_ parameter, the higher the water removal; the achieved values of WL in OD using erythritol and xylitol solutions, were more effective at higher concentrations (Figure 1a,b).

OD in inulin and oligofructose was ineffective—the observed values of WL were low (Figure 1d,e) and the values of the *k*_2_ parameter in Peleg’s equation were high (Table 4). Statistical analysis classified these values first into two homogeneous groups—with the lowest values (Table 5). This behavior was connected with the high molecular weight of these substances, which results in a low driving force of the process, while the changes in the hydrodynamic characteristics of the external phase modify the global mass transfer resistance. The evaluation of alternatives to sucrose substances (oligofructose, maltitol, and oligofructose/trehalose) was reported in research by Giannakourou and Taoukis [19]. Their results show that the highest WL during OD was in maltitol, which had the lowest molecular weight, although the effect of the alternative osmotic agents was not significant.

Mendonça et al. [31] observed that WL was significantly dependent on the duration of ultrasound pre-treatment, in the linear term for xylitol and sorbitol solutions. It was also reported that at the end of the treatments, WL was more pronounced in samples treated with solutions of sorbitol, erythritol and xylitol [29]. Lower values were obtained in samples treated with solutions of isomalt and maltitol, which are osmotic agents with lower molecular weights. 

### 2.3. Solid Gain (SG)

**Figure 2:**
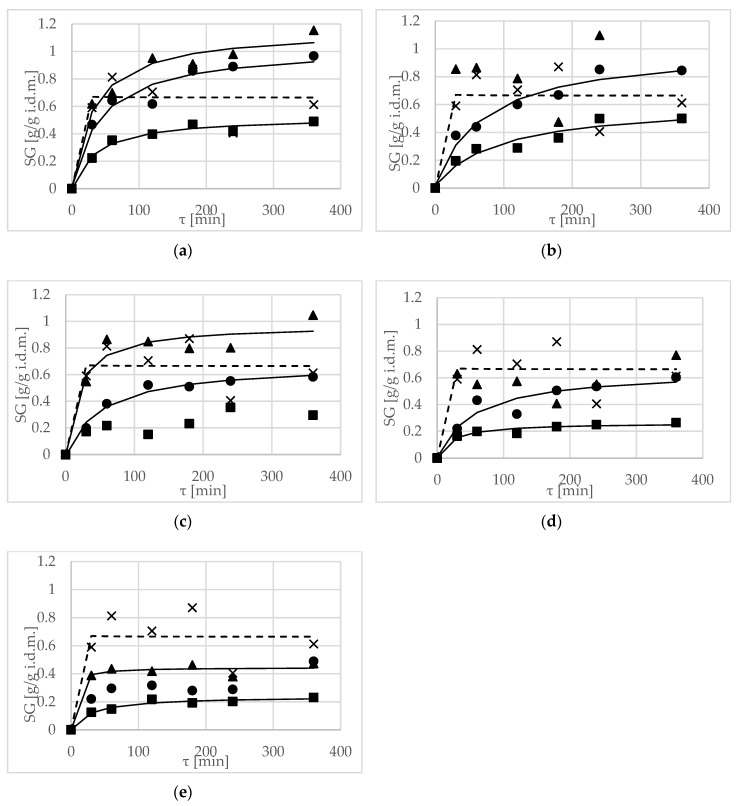
Solid gain (SG), kinetics at several concentrations, (20 (■), 30 (●), 40 (▲) and 50 (×) g/100g) at 40 °C, using different solutions: (**a**) erythritol, (**b**) xylitol, (**c**) maltitol, (**d**) inulin, (**e**) oligofructose. Lines are the Peleg’s model. The dotted line is the kinetic reference for sucrose.

During the process of OD, the main phenomenon is water loss. SG in the material was inconsiderable, with maximum values of approximately 1 g/g i.d.m. when using 40% solution concentrations of xylitol, erythritol and maltitol, as well as 30% solution concentrations of xylitol and erythritol, in durations ranging from 4 to 6 h (Figure 2a–c). Mendonça et al. [29] observed a similar situation at the end of the process; the highest SG in yacon roots was obtained with erythritol solution.

Solution concentration had a significant influence on SG during the process (Table 7). As with the parameters discussed above, greater efficiency than sucrose was reported when 30 and 40% solutions of erythritol and xylitol, as well as a 40% solution of maltitol, were used (Figure 2a–c). Achieved values were classified into one homogeneous group. The SG in apples, at a similar level obtained using sucrose, was also achieved in the OD process of more than 3 h in 30 and 40% solutions of inulin (Figure 2d). In the case of OD in oligofructose, the increase in dry matter in the fruit was negligible (Figure 2e), which was due to the high molecular weight of this compound. 

The lower the SG, the better the preservation of the original characteristics of the food [32]. Therefore, small values of SG connected with a high-rate of WL are desirable.

Brochier et al. [15] reported SG when they used sorbitol, glycerol and polydextrose solutions, although they did not observe any increase in dry matter using maltodextrin. The highest values of SG were achieved in the case of the first two solutions, namely sorbitol and glycerol. This was explained by their lower molecular weight compared to maltodextrin, which led to a higher osmotic pressure and easier penetration into the apple tissue. Moreover, they also pointed out that the increase of dry matter occurred mainly during the first two hours of the process. This is consistent with the results obtained in the current research, mainly with 30 and 40% erythritol solutions. 

Values of *k*_1_, *k*_2_, R^2^, χ^2^, CRV and RMSE of modelling SG using Peleg’s model are shown in Table 6. According to the data in Table 6, the *k*_1_ for all kinetic terms of mass transfer decreased with increasing concentrations of the osmotic solution, at a constant solution temperature. High values for the *k*_1_ parameter indicated a low mass transfer rate. The *k*_2_ parameter defines the equilibrium moisture content and soluble solids—achieved values were higher compared to modeling WL using Peleg’s model (i.e., the effect of water removal was smaller). Evaluating the goodness of fit of SG using Peleg’s model was different compared to modeling WL parameters. The use of Peleg’s modeling of OD was effective in all of the concentrations only in the case of the erythritol solution. This model can also be used for the prediction of SG at 20% and 30% concentrations, using xylitol and inulin, but not at the highest concentrations. In the cases of 20% maltitol and 30% oligofructose, values of the CRV parameter were more than 20%, indicating that the model cannot be used for modeling of SG during OD.

Values of SG achieved by Mendonça et al. [31] after OD of yacon tubers in 40% xylitol solution were approximately two-times higher compared to those achieved in the same conditions in a 40% sorbitol solution. In later research by Mendonça et al. [29] the SG rate was attenuated after the first hour of osmotic treatment in the 40% solution concentrations of xylitol, maltitol, erythritol, isomalt and sorbitol.

In statistical ANOVA, there was a significant influence of time on SG (Table 7). An opposite situation was observed by Fasogbon et al. [33]; in their research, solid uptake during OD of pineapple in a sugar solution had no significant change over-time, but the most significant changes in sugar/salt solution took place in the first 3 h of the OD process. Taiwo et al. [34] also reported optimal SG at 3 h for strawberry halves.

### 2.4. Water Activity (a_w_)

**Figure 3:**
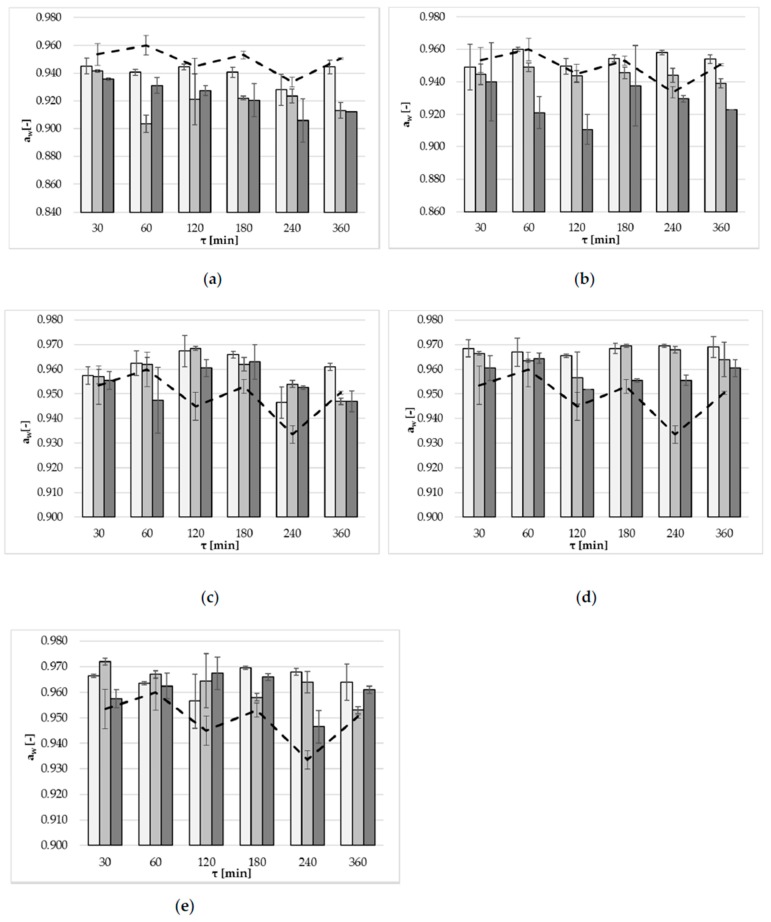
Water activity, a_w_, at several concentrations: 20 (light grey bars), 30 (grey bars), 40 (dark grey bars) g/100 g at 40 °C, using different solutions: (**a**) erythritol, (**b**) xylitol, (**c**) maltitol, (**d**) inulin, (**e**) oligofructose. The dotted line marks the values of a_w_ in a 50% solution concentration of sucrose.

A direct relationship between the increase in solution concentration, and the decrease of water activity level, was observed mainly in the case of OD using xylitol (Figure 3b). This relationship was not observed when maltitol, inulin and oligofructose solutions were used (Figure 3c–e). Statistically significant influences (α = 0.05) of a_w_ were observed of all the factors: type of osmotic agent, solution concentration, and time (Table 8). Important decreases in the values for a_w_ were observed mainly at the end of the OD process. Higher concentrations of osmotic solutions resulted in lower values of this parameter. The lowest average values were obtained for erythritol (approximately 0.928), and xylitol (0.942). This is evident in the bar graphs, which are below the line graph referring to the a_w_ of apple dehydrated in a 50% sucrose solution (Figure 3a,b). Values for a_w_ during OD in maltitol, inulin and oligofructose solutions were classified into one homogeneous group, which means that the influence of these types of osmotic solutions, on the decrease of a_w_, was not significant (Table 8). Higher values of a_w_ achieved with maltitol, inulin and oligofructose were related to small WL, compared with sucrose, as discussed above.

In the OD of yacon tubers, Mendonça et al. [31] used 40% solution concentrations of xylitol and sorbitol, and reported slightly higher values for a_w_ after OD (0.971 and 0.975, respectively). The effect of solution concentration on a_w_ was significant and negative in the linear and quadratic terms for samples treated with both solutions. In the first hour of treatment, the highest reductions of a_w_ of yacon in 40% solution concentrations were observed (erythritol: 0.936; xylitol: 0.937 and sorbitol: 0.956) [29].

The Pearson’s correlation coefficient between a_w_ and WC was calculated separately for each type of osmotic solution. In almost all cases, (except oligofructose–*p*-Value 0.117) a linear relationship between the variables was observed. The strongest relationship was observed for OD in xylitol solution (with a Pearson’ correlation coefficient of 0.81). In other cases, this relationship was weaker (erythritol: 0.67 (*p*-Value 0.000), inulin: 0.46 (*p*-Value 0.005), maltitol: 0.35 (*p*-Value 0.038)).

## 3. Materials and Methods 

### 3.1. Sample Preparation

Fresh apples, of the Paula Red variety, were purchased from a local shop. The fruits were stored at 4–5 °C and at a relative humidity of 85–90% in a refrigerator until use. Before the experiment, the apples were washed, stoned and cut into triangular slices (with the peel), with each slice 0.5 cm thick.

### 3.2. Pre-Treatment Procedure

The slices were dehydrated by OD in a water bath (Water Bath Shaker Type 357 ELPAN, Lubawa, Poland) with continuous mixing (1 Hz amplitude). The temperature of the water bath was constant. Osmotic solutions were prepared with selected substances from the polyol group: erythritol (F8030, Brenntag, Kędzierzyn-Koźle, Poland), xylitol (Brenntag), maltitol (Brenntag), as well as inulin (Frutafit CLR, Brenntag), oligofructose (Frutalose L85, Brenntag) and distilled water. Apple samples were dipped into 20, 30 and 40% concentrated syrups. To compare the OD process kinetics, a 50% sucrose solution was used as control. OD was carried out in time-ranges between 0.5 h and 6 h, at a temperature of 40 °C (atmospheric pressure), and with an approximately 2:1 syrup-to-fruit ratio. The size of a single sample was 40 ± 2 g. Afterwards, the samples were removed from the osmotic solution and blotted with absorbent paper to remove excess osmotic liquid from their surface. Two technological repetitions were performed for each treatment. 

### 3.3. Analytical Methods

#### 3.3.1. Mathematical Modeling [35]

The kinetic parameters were calculated in all of the experiments: water content (WC), solids gain (SG), water loss (WL) at different times, τ according to the following equations:

(1) $$WC=\frac{1-s_{\tau }}{s_{o}}$$

(2) $$WL=\frac{\left(1-s_{o}\right)\times m_{o}-\left(1-s_{\tau }\right)\times m_{\tau }}{s_{o}\times m_{o}}$$

(3) $$SG=\frac{s_{\tau }\times m_{\tau }-s_{o}\times m_{o}}{s_{o}\times m_{o}}$$

The model proposed by Peleg [30] was employed to fit the experimental results. In this work, WL and SG data (Equations (2) and (3)) were fitted using this Peleg’s model:

(4) $$Y=Y_{o}\pm \frac{\tau }{\left(k_{1}+k_{2}\tau \right)}$$

where parameters *k*_1_ and *k*_2_ are the known Peleg’s constants [20].

Fitting of the mathematical functions (Peleg) [30] to the experimental points was done using Table Curve 2D version 5.01 (SYSTAT Software Inc., Chicago, IL, USA). The determination coefficient (R^2^), the reduced chi-squared statistic (χ^2^), the RMSE, and the CRV, were used to evaluate the goodness of fit of the model:

(5) $$R^{2}=\frac{\sum_{i=1}^{N}{\left(MR_{i,p}-MR_{p}\right)}^{2}}{\sum_{i=1}^{N}{\left(MR_{i,e}-MR_{p}\right)}^{2}}$$

(6) $$\chi^{2}=\frac{\sum_{i=1}^{N}{\left(MR_{i,p}-MR_{i,e}\right)}^{2}}{N-n}$$

(7) $$RMSE=\sqrt{\frac{\sum_{i=1}^{N}{\left(MR_{i,p}-MR_{i,e}\right)}^{2}}{N}}$$

(8) $$CRV=100\%*\;\frac{\sqrt{X^{2}}}{Y}$$

The high R^2^ values and the lower χ^2^ and RMSE indicated that the model fits well to the experimental data. Values of CRV less than 20% indicated that the model could be used for prediction.

#### 3.3.2. Water Activity (a_w_)

Water activity is the partial vapor pressure of water in a substance, divided by the partial vapor pressure of pure water at the same temperature. Using this particular definition, pure distilled water has an a_w_ of exactly one. Water activity is an important consideration for food product design and food safety. In this study, water activity was measured using an Aqua Lab CX-2 (Decagon Devices Inc., Pullman, Washington, USA) apparatus, in accordance with the manufacturer’s instructions. The temperature of water activity determination was constant at 25 °C. Each measurement was conducted in triplicate.

#### 3.3.3. Statistical Analysis

The statistical software Statgraphics Plus version 5.1 (StatPoint), and Excel 2016 (Microsoft) were used for data analysis. The Pearson’s correlation coefficient between a_w_ and WC was calculated. The influence of pre-treatment (duration of the process, concentration, and type of osmotic solution) on dependent variables, mass transfer parameters (water loss, solid gain, the WC), as well as water activity, was evaluated by means of a multifactorial ANOVA, with a significance level of α = 0.05. In the case of significant associations, post-hoc Tukey’s test was performed. 

## 4. Conclusions

The polyols, xylitol and erythritol are suitable for use as osmotic agents in the dehydration of apples. Making these solutions provides an alternative to the use of sucrose, which is commonly used in the food industry. In the tested concentration range, maltitol solutions were too ineffective. Osmotic dehydration in solutions containing inulin and oligofructose was ineffective because of the high molecular weight of these solutions. The use of Peleg’s equation to model mass transfer kinetics during OD was effective. Increased solution concentrations from 20 to 40% indicated an increase in the initial rate of mass transfer terms and resulted in greater water removal. It is necessary to conduct further tests on the residue content of these substances in the fruit, as per the accepted limit of food content by Acceptable Daily Intake (ADI).

## Figures and Tables

**Table 1:** Water content (g/g d.m.) in apples during osmotic dehydration (OD) in different solutions.

Solution	Xylitol 20%	Xylitol 30%	Xylitol 40%	Erythritol 20%	Erythritol30%	Erythritol 40%	Maltitol 20%	Maltitol 30%	Maltitol 40%
Time [min]									
0	7.31 ± 0.01	7.31 ± 0.01	7.31 ± 0.01	7.31 ± 0.01	7.31 ± 0.01	7.31 ± 0.01	7.31 ± 0.01	7.31 ± 0.01	7.31 ± 0.01
30	5.35 ± 0.00	4.28 ± 0.01	2.56 ± 0.02	4.95 ± 0.00	3.92 ± 0.00	3.19 ± 0.00	6.03 ± 0.01	5.44 ± 0.00	3.77 ± 0.01
60	4.86 ± 0.01	3.91 ± 0.02	2.4 ± 0.03	4.31 ± 0.01	3.13 ± 0.00	2.68 ± 0.00	5.86 ± 0.01	4.73 ± 0.01	2.97 ± 0.02
120	4.54 ± 0.00	3.12 ± 0.00	2.3 ± 0.01	3.94 ± 0.00	2.87 ± 0.01	1.95 ± 0.00	6.17 ± 0.00	3.68 ± 0.01	2.82 ± 0.00
180	4.3 ± 0.00	3.05 ± 0.01	2.64 ± 0.11	3.78 ± 0.00	2.23 ± 0.01	1.8 ± 0.01	5.86 ± 0.00	4.07 ± 0.01	2.66 ± 0.00
240	3.88 ± 0.00	2.65 ± 0.00	1.97 ± 0.00	3.99 ± 0.01	2.35 ± 0.01	1.88 ± 0.03	5.5 ± 0.00	3.86 ± 0.00	2.58 ± 0.02
360	3.94 ± 0.00	2.72 ± 0.00	1.63 ± 0.03	3.74 ± 0.01	2.41 ± 0.00	1.66 ± 0.00	5.38 ± 0.00	3.56 ± 0.00	2.26 ± 0.01

**Table 2:** Water content (g/g d.m.) in apples during OD in different solutions (continued).

Solution	Inulin 20%	Inulin 30%	Inulin 40%	Oligofructose 20%	Oligofructose 30%	Oligofructose 40%	Sucrose 50%
Time [min]							
0	7.31 ± 0.01	7.31 ± 0.01	7.31 ± 0.01	7.31 ± 0.01	7.31 ± 0.01	7.31 ± 0.01	7.31 ± 0.01
30	6.6 ± 0.00	5.9 ± 0.00	3.92 ± 0.02	6.41 ± 0.01	5.75 ± 0.00	4.76 ± 0.00	3.42 ± 0.01
60	6.32 ± 0.00	4.51 ± 0.00	4.07 ± 0.01	6.51 ± 0.00	5.61 ± 0.01	4.75 ± 0.01	2.71 ± 0.00
120	6.01 ± 0.01	5.34 ± 0.01	3.8 ± 0.01	6.11 ± 0.01	5.47 ± 0.01	4.69 ± 0.00	2.39 ± 0.00
180	5.35 ± 0.00	4.94 ± 0.00	4.36 ± 0.03	6.04 ± 0.00	5.73 ± 0.00	4.59 ± 0.00	2.21 ± 0.00
240	4.91 ± 0.00	4.71 ± 0.01	4.05 ± 0.01	6.25 ± 0.00	5.61 ± 0.00	4.93 ± 0.00	2.95 ± 0.02
360	4.48 ± 0.01	4.39 ± 0.00	3.44 ± 0.01	5.67 ± 0.00	4.58 ± 0.00	4.59 ± 0.00	2.38 ± 0.01

**Table 3:** The influence of osmotic pre-treatment in different solution types, concentrations, and times, on the water content (g/g d.m.) in fruits.

Factor		*p*-Value	Contrast	+/− Limits	Difference
Type of osmotic substance	Erythritol ^a^	0.000 *	Erythritol–Inulin	0.2799	−1.8000 *
	Xylitol ^b^		Erythritol–Xylitol	0.2799	−0.3126 *
	Maltitol ^c^		Inulin–Maltitol	0.2799	0.5527 *
	Inulin ^d^		Inulin–Xylitol	0.2799	1.4874 *
	Oligo-fructose ^e^		Maltitol–Xylitol	0.2799	0.9347 *
Concentration of solution (%)	20 ^c^	0.000 *	20–30	0.1858	1.0829 *
	30 ^b^		20–40	0.1858	2.0347 *
	40 ^a^		30–40	0.1858	0.9518 *
Time (min)	30 ^d^	0.000 *	30–60	0.3205	0.4099 *
	60 ^c^		60–120	0.3205	−0.2594
	120 ^bc^		120–180	0.3205	0.0765
	180 ^b^		180–240	0.3205	0.1702
	240 ^ab^		240–360	0.3205	0.3131
	360 ^a^		360–60	0.3205	−0.8191

Statistical differences between factors; a Tukey test of main effects was performed. * denotes a statistically significant difference. Means within columns, with a different lowercase letter superscript, are significantly different (*p* < 0.05).

**Table 4:** Values of *k*_1_, *k*_2_, R^2^, χ^2^, CRV and RMSE of modeling WL using Peleg’s model.

Solution	Concentration	*k*_1_	*k*_2_	R^2^	χ^2^	CRV (%)	RMSE
Erythritol	20	7.191	0.541	0.974	0.007	5.99	0.070
	30	8.298	0.313	0.952	0.064	11.80	0.214
	40	6.511	0.240	0.986	0.027	5.88	0.139
Xylitol	20	14.245	0.621	0.954	0.007	7.54	0.071
	30	9.759	0.387	0.973	0.016	7.14	0.107
	40	3.374	0.285	0.960	0.013	4.29	0.097
Maltitol	20	-	-	-	-	-	-
	30	-	-	-	-	-	-
	40	12.225	0.341	0.981	0.014	6.32	0.101
Inulin	20	-	-	-	-	-	-
	30	-	-	-	-	-	-
	40	7.724	0.810	0.874	0.012	11.42	0.092
Oligofructose	20	-	-	-	-	-	-
	30	-	-	-	-	-	-
	40	−10.708	1.833	0.891	0.005	13.81	0.059

- denotes values of *CRV* greater than 20%, which indicates that the model cannot be used for WL prediction.

**Table 5:** The influence of osmotic pre-treatment in different types, concentrations of solutions, and time on WL (g/g i.d.m.) during OD.

Factor		*p*-Value	Contrast	+/− Limits	Difference
Type of osmotic substance	Erythritol ^e^	0.000 *	Erythritol–Inulin	0.2795	1.8406 *
	Xylitol ^d^		Erythritol–Xylitol	0.2795	0.3059 *
	Maltitol ^c^		Inulin–Maltitol	0.2795	−0.5799 *
	Inulin ^b^		Inulin–Xylitol	0.2795	−1.5347 *
	Oligo-fructose ^a^		Maltitol–Xylitol	0.2795	−0.9548 *
Concentration of solution (%)	20 ^a^	0.000 *	20–30	0.1856	−0.5202 *
	30 ^b^		20–40	0.1856	−1.3377 *
	40 ^c^		30–40	0.1856	−0.8174 *
Time (min)	30 ^a^	0.000 *	30–60	0.3205	0.1696
	60 ^ab^		60–120	0.3201	0.3097
	120 ^bc^		120–180	0.3201	−0.0518
	180 ^c^		180–240	0.3201	0.0275
	240 ^c^		240–360	0.3201	−0.1829
	360 ^c^		360–60	0.3201	0.5169 *

Statistical differences between factors; a Tukey test of main effects was performed. * denotes a statistically significant difference. Means within columns, with a different lowercase letter superscript, are significantly different (*p* < 0.05).

**Table 6:** Values of *k*_1_, *k*_2_, R^2^, χ^2^, CRV and RMSE of modeling SG using Peleg’s model.

Solution	Concentration	*k*_1_	*k*_2_	R^2^	χ^2^	CRV (%)	RMSE
Erythritol	20	67.029	1.896	0.958	0.001	7.91	0.022
	30	42.830	0.977	0.952	0.005	11.47	0.061
	40	28.254	0.868	0.945	0.005	8.98	0.058
Xylitol	20	159.927	1.677	0.910	0.002	16.07	0.041
	30	73.613	1.010	0.940	0.003	10.31	0.047
	40	-	-	-	-	-	-
Maltitol	20	-	-	-	-	-	-
	30	75.408	1.446	0.950	0.001	8.35	0.028
	40	18.773	1.025	0.896	0.010	14.27	0.084
Inulin	20	83.366	3.830	0.856	0.000	10.64	0.017
	30	87.141	1.537	0.881	0.005	18.52	0.059
	40	-	-	-	-	-	-
Oligofructose	20	123.147	4.184	0.906	0.000	9.69	0.013
	30	-	-	-	-	-	-
	40	8.971	2.250	0.951	0.001	9.18	0.028

- denotes values of *CRV* more than 20%, which indicate that the model cannot be used for prediction.

**Table 7:** The influence of osmotic pre-treatment in different types, concentrations of solutions, and time on SG (g/g i.d.m.) during OD.

Factor		*p*-Value	Contrast	+/− Limits	Difference
Type of osmotic substance	Erythritol ^d^	0.000 *	Erythritol–Inulin	0.0848	0.2609 *
	Xylitol ^d^		Erythritol–Xylitol	0.0848	0.0430
	Maltitol ^c^		Inulin–Maltitol	0.0848	−0.0924 *
	Inulin ^b^		Inulin–Xylitol	0.0848	−0.2179 *
	Oligo-fructose ^a^		Maltitol–Xylitol	0.0848	−0.1255 *
Concentration of solution (%)	20 ^a^	0.000 *	20–30	0.0563	−0.2395 *
	30 ^b^		20–40	0.0563	−0.4458 *
	40 ^c^		30–40	0.0563	−0.2064 *
Time (min)	30 ^a^	0.000 *	30–60	0.0971	−0.0936
	60 ^ab^		60–120	0.0971	0.0267
	120 ^bc^		120–180	0.0971	−0.0105
	180 ^bc^		180–240	0.0971	−0.0859
	240 ^cd^		240–360	0.0971	−0.0936
	360 ^d^		360–60	0.0971	0.2166 *

Statistical differences between factors; a Tukey test of main effects was performed. * denotes a statistically significant difference. Means within columns, with a different lowercase letter superscript, are significantly different (*p* < 0.05).

**Table 8:** The influence of osmotic pre-treatment in different types, concentrations of solutions, and time on a_w_ during OD.

Factor		*p*-Value	Contrast	+/− Limits	Difference
Type of osmotic substance	Erythritol ^a^	0.000 *	Erythritol–Inulin	0.0060	−0.0358 *
	Xylitol ^b^		Erythritol–Xylitol	0.0060	−0.0140 *
	Maltitol ^c^		Inulin–Maltitol	0.0060	0.0059
	Inulin ^c^		Inulin–Xylitol	0.0060	0.0218 *
	Oligo-fructose ^c^		Maltitol–Xylitol	0.0060	0.0159 *
Concentration of solution (%)	20 ^c^	0.000 *	20–30	0.0040	0.0073 *
	30 ^b^		20–40	0.0040	0.0132 *
	40 ^a^		30–40	0.0040	0.0059 *
Time (min)	30 ^b^	0.014 *	30–60	0.0068	0.0035
	60 ^ab^		60–120	0.0068	−0.0006
	120 ^ab^		120–180	0.0068	−0.0029
	180 ^ab^		180–240	0.0068	0.0057
	240 ^a^		240–360	0.0068	0.0001
	360 ^a^		360-30	0.0068	0.0070 *

Statistical differences between factors; a Tukey test of main effects was performed. * denotes a statistically significant difference. Means within columns with a different lowercase letter superscript, are significantly different (*p* < 0.05).

## Nomenclature

OD – osmotic dehydration,
WC – water content, (g/g d.m.)
WL – water loss, (g/g i.d.m.)
SG – solids gain, (g/g i.d.m.)
*m* – sample mass, (g)
*s* – dry solids mass of material, (g)
*τ* – time of osmotic dehydration, (min)
*k*_1_ – parameter of Equation (4), (kg/kg)
*k*_2_ – parameter of Equation (4), (kg/kg·h)
MR_i,p_ – predicted dimensionless moisture ratio
MR_i,e_ – experimental dimensionless moisture ratio
N – number of observation
n – number of constants in the model equation
Y – mean experimental value of WL, SG or WC, respectively [in Equation (6)]
Subscripts:
o – initial
τ – time (min)
